# Size Influences on the Survival of Willow Cuttings Under Operational Field Conditions

**DOI:** 10.1002/ece3.70835

**Published:** 2025-01-09

**Authors:** Alan Pollock, Kyle R. Grant, Amanda Schoonmaker

**Affiliations:** ^1^ Northern Alberta Institute of Technology Edmonton Alberta Canada; ^2^ Northern Alberta Institute of Technology Centre for Boreal Research Peace River Alberta Canada

**Keywords:** adventitious rooting, cutting size, outplanting, pre‐emergence variation, reclamation, restoration, *Salix*, shoot storage

## Abstract

Willows (genus *Salix*) are increasingly used in operational‐scale ecosystem reclamation; however, different opinions exist regarding the optimal cutting size for planting under field conditions. We compared the survival of field‐planted willow cuttings sourced from upland and lowland areas with varying diameters and lengths across two growing seasons. Cuttings were grouped into 15 size classes with different diameters (0.5, 1.0, 2.0, and 3.0 cm) and lengths (15, 30, 50, and 100 cm) and planted in groups according to their source (upland or lowland, each area potentially reflecting a different assemblage of species) within three reclaimed industrial borrow pits east of Peace River, Alberta. We considered cuttings that displayed leaf flushing as surviving individuals. Survival probability tended to be greater for larger diameter and length cuttings sourced from lowland habitats. Cutting survival was greatly reduced in year two, especially for upland‐sourced cuttings. The greater survival in larger sizes we observed may be due to greater total carbohydrates available for leaf flushing and rooting. As we did not control for species composition, the higher survival of cuttings from low‐lying areas may result from species‐specific differences among cutting sources, reflected by compositional differences we detected among surviving individuals in year two. Our findings suggest that under field settings, cuttings with > 2.0 cm diameter by 50–100 cm length sourced from low‐lying flood‐prone areas may be optimal for willow establishment. Further studies should examine the role of species and population‐level genetics in driving the upland versus lowland differences in observed willow‐cutting survival.

## Graphical abstract

This study examined the effect of source location (where willow cuttings were collected from) and the size of cuttings on the short‐term survival of these cuttings.

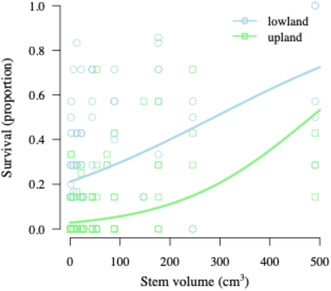

## 1 Introduction

Willows (genus *Salix*) are ubiquitous, fast‐growing pioneer species that thrive across a wide range of conditions, from dry to moist forests, floodplains, and wetlands to areas along lakeshores and streams. They are particularly well adapted to suboptimal soils (Argus 1986). Willows serve various functional roles, including stabilizing soils (Gray and Sotir 1996), enhancing soil biological properties (see Baum et al. 2009; Stauffer et al. 2014), and providing food and habitat for wildlife species (Göransson 1994; see Haeussler, Coates, and Mather 1990) and pollen for pollinators (Reddersen 2001; Ostaff et al. 2015). The ability of willows to persist under a wide range of environmental conditions combined with their deep root systems and fast growth rates has also made them suitable for the phytoremediation of heavy metal polluted soils (Greger and Landberg 1999; Kuzovkina and Volk 2009; Pilipović et al. 2019). These properties make willows desirable species for the reclamation of degraded sites, industrial spoils, mine and gravel pits, peatlands, overburdens, quarries, and highly eroded soils (see Kuzovkina and Volk 2009). There has thus been a growing interest in the use of willows in reclamation and ecological engineering projects throughout North America and Europe (Kuzovkina and Volk 2009).

There is a range of opinions regarding the appropriate stem‐cutting size for the vegetative propagation of willows. Burgess, Hendricksona, and Roya (1990) found that cutting size increased the survival of white willows (*Salix alba* L.) under nursery conditions, with cutting length being more important than diameter. Cuttings with diameters greater than 1.3–1.9 cm and lengths of 22.9 cm had no further improvement in survival. Rossi (1999) found better survival and growth among longer cuttings planted within abandoned agricultural fields, up to 30–50 cm in length. A greenhouse experiment by Greer, Pezeshki, and Shields Jr (2006) found that 1 cm diameter black willows ( *S. nigra* Marshall) had 100% survival within three moisture conditions (control, drought, and periodic flooding) compared to 5 and 10 cm diameter cuttings (although the 10 cm diameter cuttings also had 100% survival within the periodically flooded treatment). Under periodic flooding, 10 cm diameter cuttings had greater leaf numbers, weight, and area; greater height growth; and root and shoot weights, suggesting that different willow cutting sizes may be appropriate under different planting conditions. However, a general increase in willow cutting size (length and diameter) has been associated with greater increases in shoot biomass (Burgess, Hendricksona, and Roya 1990; Rossi 1999; Verwijst et al. 2012), likely due to more available stored carbohydrates in larger cuttings (Carpenter, Pezeshki, and Shields 2008).

Willow cutting survival post‐planting varies by species and genotypes (Densmore and Zasada 1978; Mosseler and Major 2014; Mosseler, Major, and Labrecque 2014). Riparian willows, which can persist under flood‐prone environments, are more likely to develop adventitious roots (Densmore and Zasada 1978), critical for survival and growth in wet situations. For example, two riparian willow species ( *S. alaxensis* (Andersson) Coville and *S. myrtillifolia* Andersson) and three non‐riparian ( *S. bebbiana* Sarg, *S. scouleriana* Barratt ex Hook., and *S. glauca* ) were tested for rooting abilities in both a controlled greenhouse situation and in the field, using 19 cm cuttings; the riparian species rooted quite readily along the entire length of the submerged portion of their stems, whereas very few of the non‐riparian species produced roots. When non‐riparian species did produce roots, they were found only at the basal end of the cuttings. This feature in riparian species may be an adaptation to being flattened and uprooted in flooded environments, where the ability to produce roots from broken‐off or flattened stems is advantageous for survival and dispersal (Densmore and Zasada 1978).

Operational field conditions pose challenges for willow cutting establishment; for example, cuttings may suffer losses from vegetative competition, must be thick enough to withstand physical forces acting upon them, and have sufficient stored carbohydrate energy to sprout roots and shoots. Limited investigation has been conducted to determine how pre‐planting variation in willow cuttings, including size (length and diameter) and source location, influence willow establishment under operational field conditions. We aimed to evaluate the effects of these two factors on the short‐term survival of willow cuttings, given that they are sources of pre‐planting variation that could easily be controlled by practitioners. We tested the survival of 15 different size combinations of mixed‐species willow stem cuttings sourced from either upland or lowland flood‐prone areas within reclaimed industrial borrow pits and identified the species present among surviving cuttings sourced from both areas after two growing seasons.

## 2 Methods

### 2.1 Study Region

Study sites and willow cutting collection locations were located approximately 50 km NE of Peace River, Alberta, within the central mixedwood natural subregion of Alberta, Canada (Table 1). Soil groups in this subregion are dominated by gray luvisols and organics (Alberta Agriculture, Food and Rural Development 1995), reflecting a mixture of upland and wetland sites. Total precipitation for this area in 2013 was 515 mm with an annual average air temperature of 1.6°C. In 2014, total precipitation was 376 mm with an average annual air temperature of 2.3°C (Alberta Climate Information Services, https://acis.alberta.ca/township‐data‐viewer.jsp). Essentially, the trial was established in a cooler‐wetter year, while the second year reflected drier‐warmer conditions for the region.

**TABLE 1:** (a) Locations of field collection sites where willow cuttings were harvested and study sites where willow cuttings were planted. (b) plot‐level details at each borrow pit (BP) site summarizing the aspect when plots were placed on a sloped position or level when the slope grade was minimal.

(a)			
Site	Elevation	GPS coordinates	Description of location
Collection locations			
1	690	56.277587° −116.584653°	Lowland location adjacent to an industrial well pad. This area was dominated by *Salix* spp., *Alnus incana* and *Betula pumila* .
2	698	56.302272° −116.544530°	Upland location, where land had been cleared in a 5 m wide line (pipeline construction). The collection site was dominated by regenerating *Salix* spp., *Populus balsamifera* , and *Populus tremuloides* . It was immediately adjacent to mature *P. tremuloides* and *Picea glauca* forest.
3	673	56.308419° −116.567584°	Upland location, within a right of way (cleared of trees several years prior for construction of a powerline). This location was immediately adjacent to mature *Pinus bankiana* forest.
4	670	56.312613° −116.567259°	Lowland location, directly north of collection site #3 in a wetter area dominated by *Salix* spp., *Alnus incana* , and *Betula pumila* .
Study site locations			
Yellow borrow pit	673	56.325748° −116.580039°	Adjacent to an operating well pad on the south edge is cleared land with a gravel road to the southwest. The remaining perimeter was surrounded by mature *P. tremuloides* and *Picea glauca* forest. The site was 1.3 ha in size.
Pink borrow pit	696	56.294877° −116.558758°	Adjacent to a gravel road with a 35 m treed buffer separating the road from the site; otherwise surrounded by mature *P. tremuloides* and *Picea glauca* forest. The site was 0.8 ha in size.
Blue borrow pit	674	56.344920° −116.652521°	Adjacent to a gravel road with a 34 m treed buffer separating the road from the site. There was an older harvest block on the west edge with the remaining perimeter surrounded by mature *P. tremuloides* and *Picea glauca* forest. The site was 1.1 ha in size.

(b)			
	Blue BP	Yellow BP	Pink BP
Lowland plot 1	North	Northeast	Level
Lowland plot 2	West	Level	Level
Lowland plot 3	West	Level	West
Upland plot 4	North	East	West
Upland plot 5	West	Level	Level
Upland plot 6	West	Level	Level

### 2.2 Collection of Plant Material

In April 2013, over 4 days, we collected willow cuttings of 1.0–1.5 m lengths at four locations within the vicinity of the future study sites (Table 1). Cuttings were collected from two distinct site types, hereafter referred to as lowland and upland sites (Table 1); this sampling was intended to capture a distinct compositional differences in species, as it was not feasible to accurately identify *Salix* to species during winter collection. Lowland locations were characterized as those with periodic inundation and the presence of *Carex* and other woody species associated with treed swamp and fen communities (e.g., *Larix laricina* and *Betula pumila* ). Upland locations were in more well‐drained landscapes within openings adjacent to mixedwood boreal forests (dominated by *Populus tremuloides* and *Picea glauca* ). The sampling of cuttings was intended to reflect an operationally implemented collection where individual stems were collected across many individual plants (and ultimately species). All cuttings were pooled into lowland or upland site collections and placed in a dark, cold storage at −4°C until processing and planting. Altogether, 470 upland‐origin and 490 lowland‐origin cuttings were harvested, with most cuttings further processed ahead of planting into size classes as described in the subsequent section.

We removed the cuttings from cold storage in June and recut the bottom ends to facilitate water uptake. The cuttings were sorted into four diameter (0–0.5, 0.5–1.0, 1.0–2.0, and 2.0–3.0 cm) and four length (15, 30, 50, and 100 cm) classes where the diameter was determined at the midpoint of the cutting. Diameter and length classes were crossed (potential for 16 combinations); however, the 0–0.5 diameter × 100 cm length class was not practically achievable, which resulted in a total of 15 size class combinations. To distribute enough cuttings across the size classes, cuttings were further processed (cut into new lengths) to meet the size specifications. Due to the morphology of the species collected, no cuttings fit into the 0.5 cm diameter by 100 cm length class. All cuttings were immersed in buckets of water for 24 h prior to field planting, and the terminal ends were painted with non‐toxic white latex paint to reduce water loss.

### 2.3 Experimental Layout

We established eighteen replicate plots across three recently reclaimed industrial borrow pits in June 2013. The distances between the two furthest borrow pits (Blue and Pink sites) were 9 km, with one borrow pit centered approximately halfway between the other two (Table 1). The willow cutting collection sites were located between the central and southernmost borrow pits. A borrow pit is a type of disturbance from which soils (typically clay‐rich) are removed for use in road or well site construction (Scrafford, Nobert, and Boyce 2020). After excavation activities are completed, these sites are recontoured to reduce slope angles, and salvaged topsoil (mixture of forest floor and A soil horizons) and subsoil (B horizon) will be respread around the banks of the open excavation to facilitate a functional and usable water feature. Borrow pits are generally small (1–2 ha in size) with undisturbed forest or other industrial features surrounding 50%–75% of the site. Due to the relative proximity to open water and generally fine‐textured, clay‐rich soils, these sites often support a diversity of riparian and wetland habitats and have been documented as high‐use habitats for beavers ( *Castor canadensis* ) (Scrafford, Nobert, and Boyce 2020). In the present investigation, final recontouring and soil replacement activities were completed the winter before planting.

At each borrow pit, we established three plots comprised of cuttings taken from upland areas and three from lowland areas. Plots were placed adjacent to the central water feature located at each borrow pit. The edges of individual plots were 1–10 m from the high‐water line of these water features. Each plot contained 15 rows with seven cuttings established 1 m apart within each row (plots were 14 × 6 m in size). Each row contained one diameter by length size class as plots were oriented parallel with the water body. This design choice ensured that all size classes within a single plot experienced the same range of potential soil moisture conditions.

**FIGURE 1:**
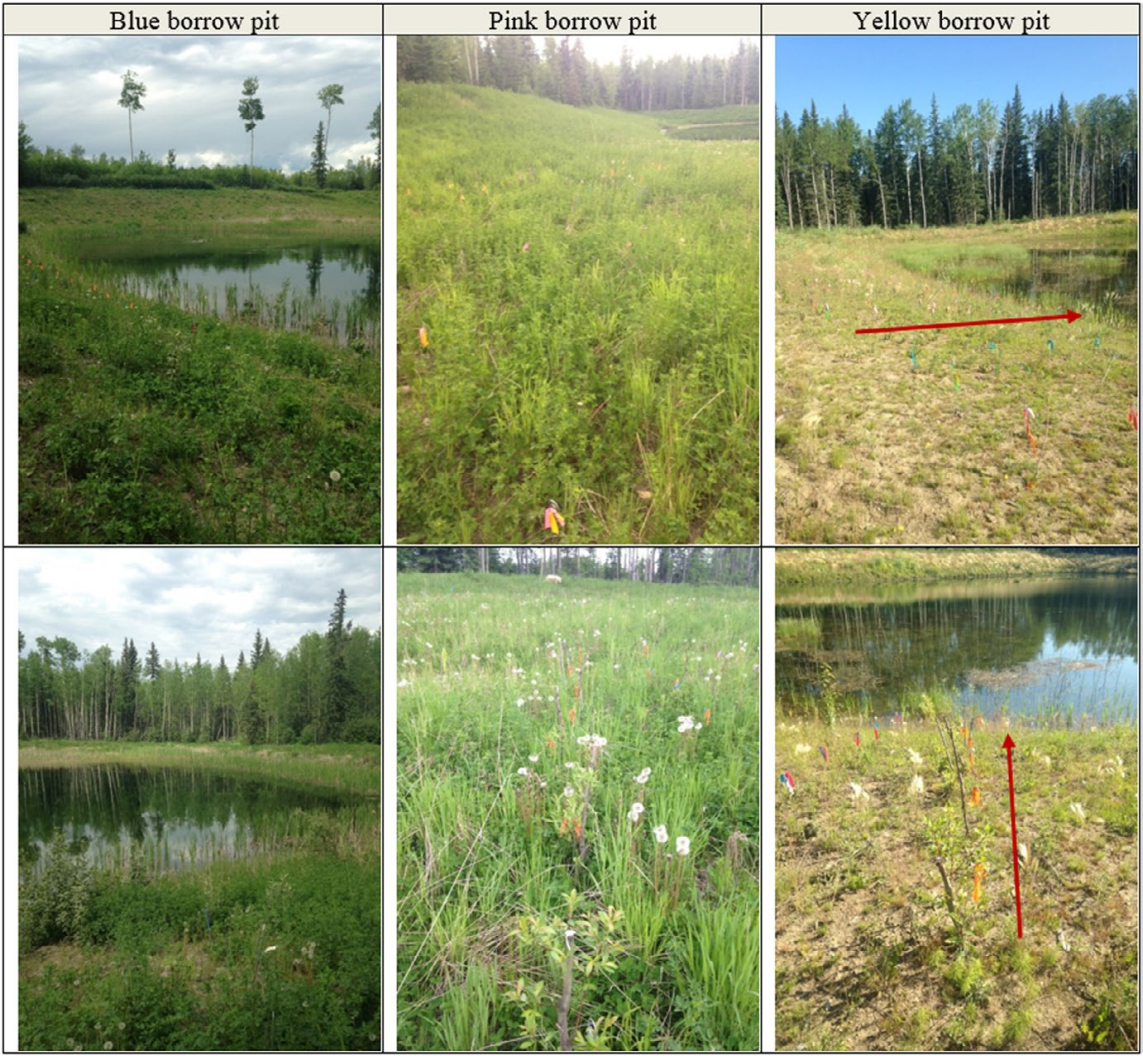
Images illustrating variation between borrow pit locations and plot layout. Images taken between June 22 and July 21, 2014. The red arrows illustrate the direction of planted rows of cuttings within a single size class where each row had a different flagging tape combination applied to reflect a specific size class.

While we aimed to establish cuttings of all 15 size classes in each plot, the availability of plant material in specific size classes resulted in some plots containing an incomplete mixture of classes (Table 2). Cutting‐size classes were randomly distributed within each plot with 1‐m spacing between each cutting (see Figure 1 for a visual example of plots). Cuttings were planted to a minimum depth of 30 cm using planting bars in May 2013.

**TABLE 2:** Detailed summary of the number of individual cuttings established within replicate plot locations organized by length and diameter class. Each plot was originally intended to contain 7 cuttings of each length by diameter class, though some plots contained fewer or no individuals in cases where plant material was limited. Volume reflects the midpoint of each diameter class, with values in brackets representing the upper and lower boundaries of the diameter class.

Length class (cm)	Diameter class (cm)	Volume (cm^3^)	Blue borrow pit							Pink borrow pit						
			Total	Lowland plots			Upland plots			Total	Lowland plots			Upland plots		
				1	2	3	4	5	6		1	2	3	4	5	6
15	0–0.5	1 (0–3)	35	7	7	7	7	7	0	41	7	7	6	7	7	7
	0.5–1.0	7 (3–12)	39	7	4	7	7	7	7	35	7	0	7	7	7	7
	1.0–2.0	27 (12–47)	14	7	0	0	0	0	7	35	0	7	7	7	7	7
	2.0–3.0	74 (47–106)	0	0	0	0	0	0	0	21	0	0	0	7	7	7
30	0–0.5	1 (1–6)	42	7	7	7	7	7	7	42	7	7	7	7	7	7
	0.5–1.0	13 (6–24)	42	7	7	7	7	7	7	42	7	7	7	7	7	7
	1.0–2.0	53 (24–94)	14	0	0	0	7	0	7	35	0	7	7	7	7	7
	2.0–3.0	147 (94–212)	0	0	0	0	0	0	0	14	0	0	7	7	0	0
50	0–0.5	2 (2–10)	21	7	7	0	7	0	0	42	7	7	7	7	7	7
	0.5–1.0	22 (10–39)	35	7	7	7	0	7	7	42	7	7	7	7	7	7
	1.0–2.0	88 (39–157)	14	6	0	1	0	0	7	42	7	7	7	7	7	7
	2.0–3.0	245 (157–353)	0	0	0	0	0	0	0	31	0	3	7	7	7	7
100	0.5–1.0	44 (20–79)	29	7	1	0	7	7	7	42	7	7	7	7	7	7
	1.0–2.0	177 (79–314)	21	7	0	0	7	7	0	35	7	7	7	0	7	7
	2.0–3.0	491 (314–707)	0	0	0	0	0	0	0	42	7	7	7	7	7	7
Grand total			306	69	40	36	56	49	56	541	70	80	97	98	98	98

Length class (cm)	Diameter class (cm)	Volume (cm^3^)	Yellow borrow pit						
			Total	Lowland plot ID			Upland plot ID		
				1	2	3	4	5	6
15	0–0.5	1 (0–3)	35	7	0	7	7	7	7
	0.5–1.0	7 (3–12)	42	7	7	7	7	7	7
	1.0–2.0	27 (12–47)	14	0	0	0	0	7	7
	2.0–3.0	74 (47–106)	0	0	0	0	0	0	0
30	0–0.5	1 (1–6)	28	7	0	7	7	0	7
	0.5–1.0	13 (6–24)	42	7	7	7	7	7	7
	1.0–2.0	53 (24–94)	25	0	4	0	7	7	7
	2.0–3.0	147 (94–212)	7	0	0	0	7	0	0
50	0–0.5	2 (2–10)	42	7	7	7	7	7	7
	0.5–1.0	22 (10–39)	41	7	7	7	7	7	6
	1.0–2.0	88 (39–157)	35	0	7	7	7	7	7
	2.0–3.0	245 (157–353)	0	0	0	0	0	0	0
100	0.5–1.0	44 (20–79)	42	7	7	7	7	7	7
	1.0–2.0	177 (79–314)	35	7	7	7	7	0	7
	2.0–3.0	491 (314–707)	13	7	6	0	0	0	0
Grand total			401	63	59	63	77	63	76

### 2.4 Measurements and Species Identification

Survival was assessed after the first growing season (September 2013) and second growing season (September 2014) by tallying the cuttings that displayed leaf flushing and had existing live tissues (i.e., green leaves and stems). At the conclusion of the trial, living individuals were identified to the species level to identify the species composition of survivors (relative abundance) among upland and lowland cutting sources.

### 2.5 Statistical Analysis

All statistical analyses were performed with R statistical programming software (R Core Team 2024). A generalized linear mixed‐effects model (GLMM) was developed using the function *glmmTMB* from the R package *glmmTMB* (Brooks et al. 2017) for each of the 2 years of assessment. Survival was fitted using a binomial distribution (family = binomial, link = logit) with four fixed effects factors, including the cutting origin (lowland or upland), diameter and length classes, as well as the position within the plot (planting position). Planting position was treated as an ordinal factor reflecting the relative proximity of individual cuttings to open water (position 1 was furthest away and position 7 closest). The two‐way interactions between cutting origin and all other factors were included in the final model; however, due to insufficient replication, we did not assess the three‐ or four‐way interactions of fixed effects. Random effects of this model were hierarchical, with planting line position nested within plot and plot nested within the borrow pit location. This structure in the random effects reflects how a group of 7 cuttings was planted along single lines within an experimental plot. Mean estimates and confidence intervals were determined using the *emmeans* R package (Lenth 2018). When significant differences in main effects (or their interactions) were detected (*p* ≤ 0.05), pairwise comparisons between treatments were assessed with post hoc multiple comparison tests (Tukey tests) using the *emmeans* function (Lenth 2018). Model assumptions were assessed with diagnostic plots of fitted and residual values and histograms of residuals using the *DHARMa* R package (Hartig 2022).

To combine the effects of diameter and length, we also examined the relationship between cutting volume and collection origin on 2‐year survival. We similarly employed a GLMM where cutting volume was treated as a continuous variable and collection origin a categorical variable. The random effects structure followed the same approach as above, with planting line nested within plot and plot nested within the borrow pit location. Stem volumes were calculated as a cylinder with diameter measured at the midpoint of the cutting. Although these volumes are not precise, we were only interested in the relative relationships between larger and smaller cuttings. Model assumptions were assessed with diagnostic plots of fitted and residual values and histograms of residuals using the *DHARMa* R package (Hartig 2022).

To explore potential differences among plots and borrow pit locations, a similar modeling and testing approach (as described above) was also implemented within a subset of cutting data, which included all length classes but only the 0.5–1.0 cm diameter class, as the number of mixtures of cuttings at each borrow pit and plot was unbalanced (Table 2). To evaluate differences among plots, two fixed effects were included in the model: plot ID and cutting length, where cutting length was included given it was known to be influential in explaining differences in survival. The borrow pit location was used as a random effect for this simpler model. To examine patterns in survival associated with borrow pit locations, a similar two‐factor model was also implemented and included borrow pit location and cutting length, with plot ID considered a random effect. As this exploration was not the primary goal of the study, results of these analyses are provided within the Appendix S1.

## 3 Results

The arithmetic proportion of living cuttings after the first year was 0.47, and after the second year was 0.20. The results below illustrate the results of formal statistical analyses and effects due to cutting size and planting location.

### 3.1 Survival by Cutting Size

**FIGURE 2:**
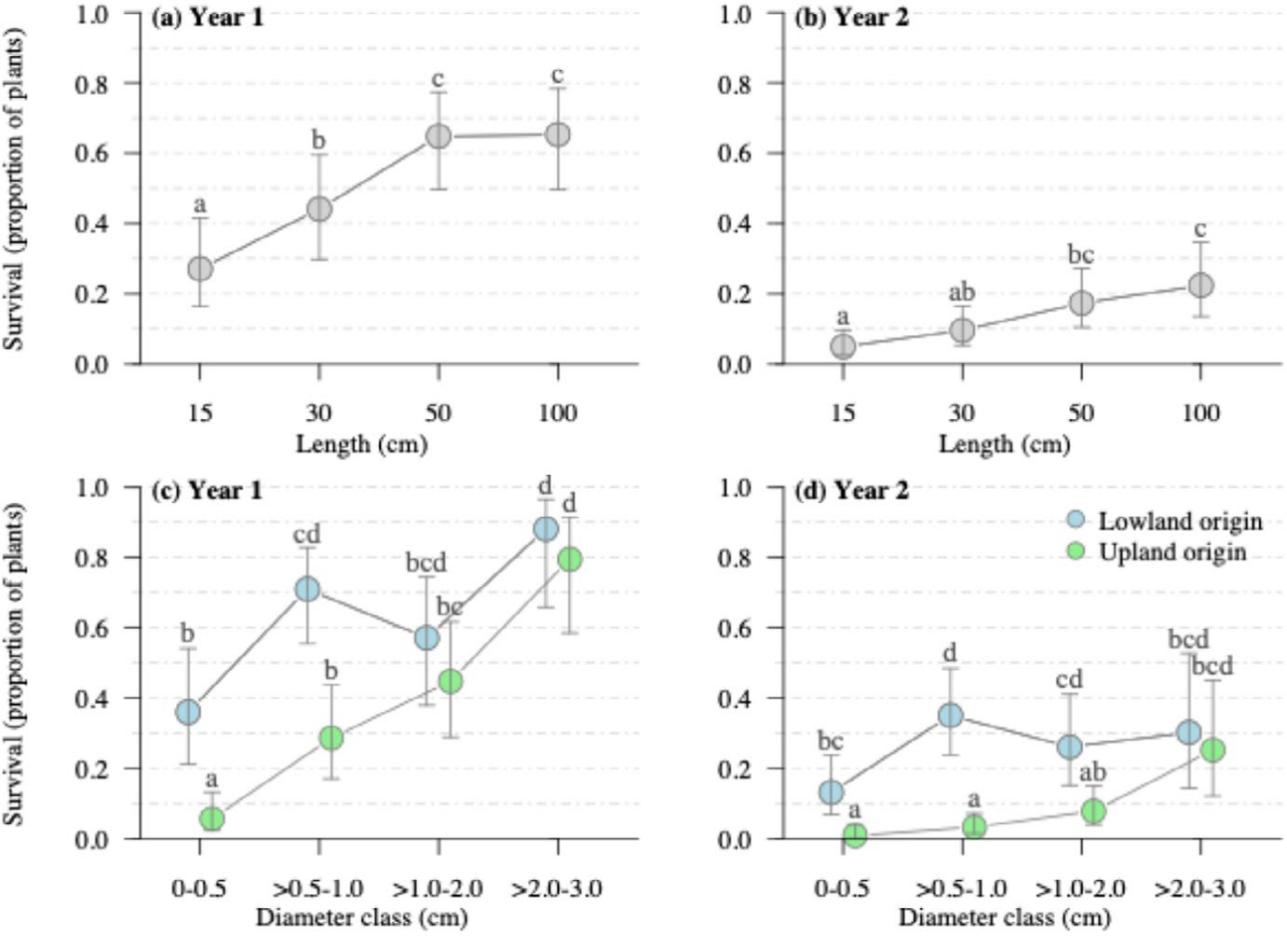
Estimated marginal mean survival (expressed as a proportion) in year 1 (2013) and 2 (2014) of willow cuttings collected from lowland and upland locations grouped by cutting length (a, b) and diameter (c, d). Error bars represent 95% confidence intervals (*n* = 9). Significant differences among means are indicated by differing letters.

After the first growing season, the length of cuttings (*p* < 0.0001) and the interaction between source location (*p* < 0.0001) and cutting diameter (*p* = 0.008) were significant factors associated with cutting survival, though the planted position of cuttings was not significant (*p* = 0.4991) (Table 3). The 15 cm cutting length class was associated with significantly lower proportional survival at 0.30 on average; the 30 cm length class was 0.40, and the 50–100 cm length class averaged 0.65 (Figure 2a). For lowland‐sourced cuttings, only the smallest diameter class (0–0.5 cm) resulted in a significant decline in survival at 0.40 compared with 0.6–0.9 in larger diameter classes (Figure 2c). In contrast, for upland‐sourced cuttings, there was a progressive increase in survival associated with increased diameter size class, where the smallest diameter class was 0.10 and the largest 0.80, on average (Figure 2c). The two smallest diameter classes showed higher survival from lowland sources compared with upland sources, while there was no effective difference for the larger diameter classes in the first year (Figure 2c).

**TABLE 3:** Generalized linear mixed‐effects model output for willow cutting survival.

Year of assessment	Factor level	Chi‐square value	Degrees of freedom	*p*
Year 1 (2013)	Location	20.35170	1	< 0.0001
	Diameter	68.54210	3	< 0.0001
	Length	47.05750	3	< 0.0001
	Planted position	5.35510	6	0.4991
	Location × Diameter	11.82110	3	0.0080
	Location × Length	1.48190	3	0.6865
	Location × Planted position	12.37150	6	0.0542
Year 2 (2014)	Location	43.48780	1	< 0.0001
	Diameter	28.34310	3	< 0.0001
	Length	33.21810	3	< 0.0001
	Planted position	10.88850	6	0.0919
	Location × Diameter	15.64810	3	0.0013
	Location × Length	4.01010	3	0.2604
	Location × Planted position	2.19400	6	0.9010

*Note:* Factors tested included the source where cuttings were collected (location), the size of the cutting (length and diameter), planted position relative to the water source, as well as interactions between location and diameter, location and length, and location and planted position. Interactions between diameter and length could not be tested due to incomplete crossing of these factors. Survival data was fit with a binomial distribution. Factors were considered significant at ∝ < 0.05.

After two seasons, there was still a significant positive relationship between survival and initial cutting length (*p* < 0.0001, Table 3), with the highest survival in 50–100 cm lengths and the lowest survival in the 15–30 cm length class (Figure 2b). Similar to year 1, there continued to be a significant interaction between source location and diameter (*p* = 0.0013; Table 3). This interaction resulted from differences in survival patterns between lowland and upland cuttings, where lowland cuttings had similar survival in the 3 largest diameter classes and only a significant decline in the 0–0.5 cm diameter class (Figure 2d). In contrast, for upland‐sourced cuttings, there was no significant improvement in diameter except for the largest diameter class > 2.0 cm (Figure 2).

**FIGURE 3:**
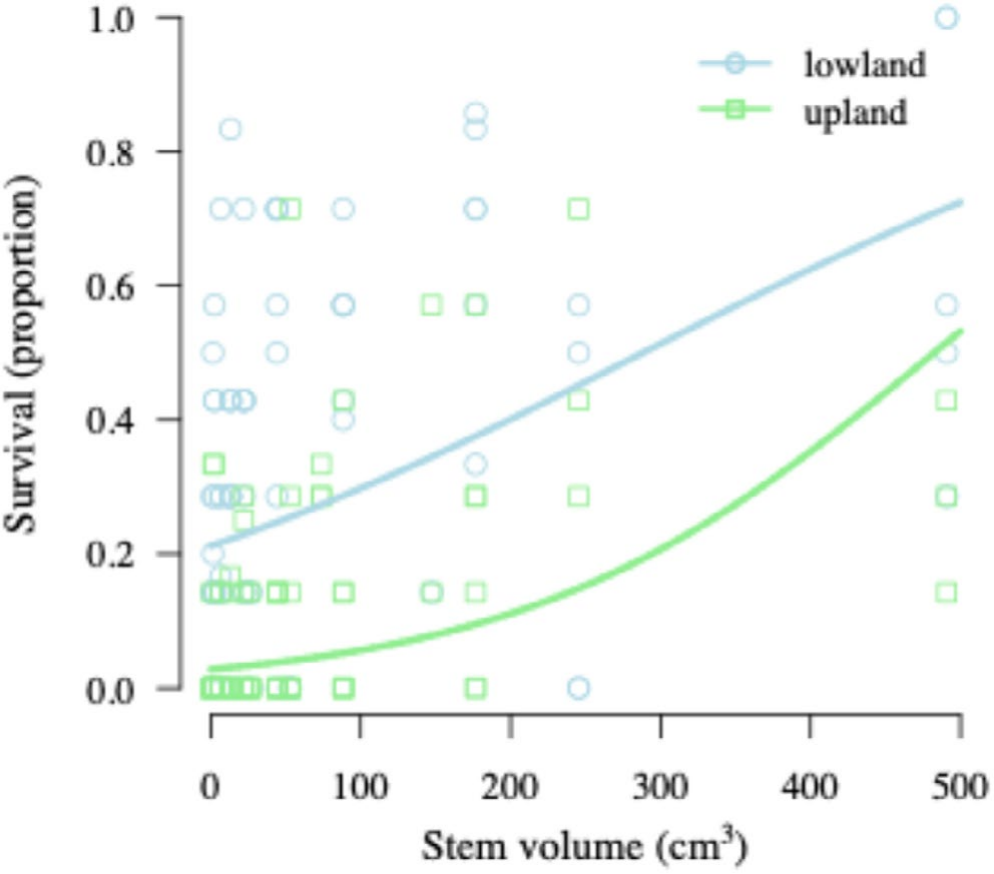
Probability of survival of willow cuttings collected from upland or lowland locations across a range of cutting volumes after two growing seasons. The solid line indicates the fitted model estimate predicting survival across a range of willow cutting volumes. Points indicate survival of cuttings averaged over a single line to reflect the variation in proportional survival across plot data. Refer to Table 4 for coefficient estimates.

Examining proportional survival after two growing seasons of cutting by volume through generalized linear regression illustrated many of the same broad trends as the diameter and size classes. Significant fixed effects included cutting volume and source location, though there was no significant interaction between these effects (Table 4). For any given stem volume, lowland‐sourced cuttings showed higher proportional survival (Figure 3). The shape of the fitted line for both sources showed continual improvement in proportional survival by volume (Figure 3).

**TABLE 4:** Estimates of binomial model coefficients for prediction of 2‐year probability of survival of upland and lowland origin cuttings.

Parameter	Estimate	95% Confidence interval	*p*
Intercept	−1.314	−1.9000 to −0.7283	< 0.0001
Volume	0.0045	0.0020 to 0.0071	< 0.0001
Upland source	−2.2388	−2.9528 to −1.5249	< 0.0001
Volume × Upland source	0.0028	−0.0013 to 0.0069	0.1757

*Note:* Fixed effects included the volume of cutting (continuous variable) and source location of cutting material (categorical variable).

### 3.2 Survival by Planting Position—Relative Proximity to Open Water

There was no difference in willow cutting survival associated with individual cuttings' relative proximity to open water (planting position) after the first (*p* = 0.4991) or second (*p* = 0.0919) growing season (Table 3). Similarly, the interaction between cutting sampling location and planting position was not significant in year 1 (*p* = 0.0542) or year 2 (*p* = 0.9010) (Table 3).

### 3.3 Species Composition

**FIGURE 4:**
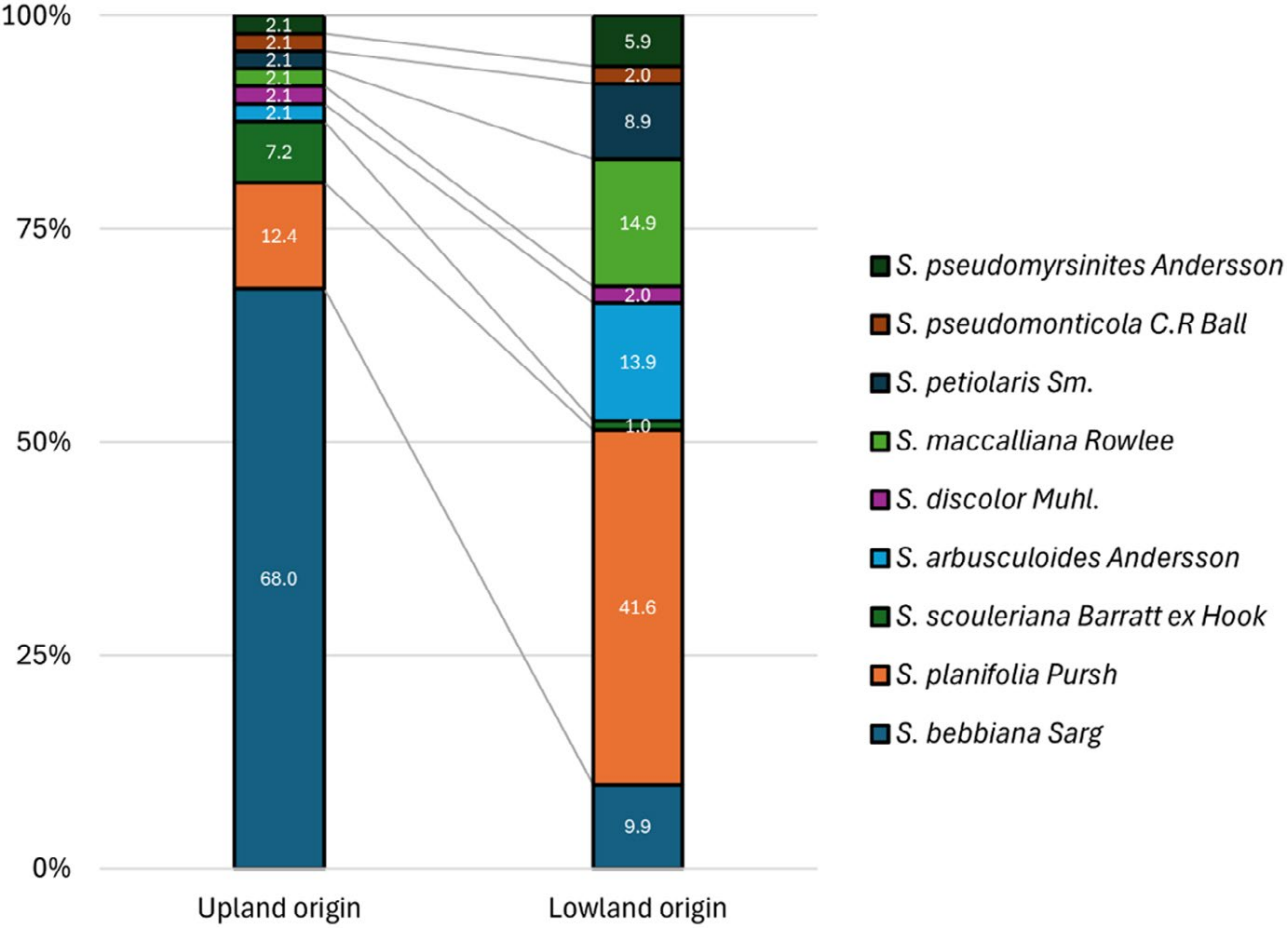
Relative abundance (%) of surviving willow (*Salix*) species identified following the second growing season belonging to upland and lowland origin.

A total of nine willow species were identified after the second year, with all species identified represented in both the lowland and upland origin plant material (Figure 4), with the same two species ( *S. bebbiana* and *S. planifolia* ) together comprising 50% or more of the individuals in lowland and upland origin locations. Within the upland origin cuttings, the non‐riparian species *S. bebbiana* had the highest relative abundance among survivors at 68.0%, followed by *S. planifolia* at 12.4% and *S. scouleriana* at 7.2%. The remaining six species each accounted for ~2% of survivors (Figure 4). The highest lowland survivors were of the riparian species *S. planifolia* at 41.6%, with *S. maccalliana* at 14.9% *S. arbusculoides* at 13.9%, and the remaining proportion of survivors more evenly distributed among species (Figure 4).

## 4 Discussion

Our findings indicate that larger willow cuttings than previously reported through nursery studies may be beneficial for increasing survival under non‐cultivated mixed‐species field‐planting conditions. Further, the tendency for larger cutting sizes to have greater survival was weaker among upland‐sourced cuttings, particularly during the second growing season. In this study, it is likely that the collection source is reflective of different compositional availabilities of willow species, where the lowland source may have comprised a greater abundance of species with higher vegetative growth capacity than those from the upland sources. While there was a progressive survival benefit associated with the largest volume cuttings in this study, these extremely large cuttings may be impractical to handle and collect operationally. Interestingly, the relative proximity to water was not an important factor for cutting survival, at least within the small experimental plots (6 m distance between the ‘driest’ and ‘wettest’ positions) these cuttings were evaluated under.

Although we found the same species among surviving cuttings sourced from both upland and lowland areas, the species composition (relative abundance) was distinct for each source, providing a potential explanation for differences in survival among source locations. Willow species are known to differ in their ability to produce adventitious roots (Talbot, Etherington, and Bryant 1987), with greater rooting ability among species that grow in low‐lying or riparian areas (Densmore and Zasada 1978; Mosseler, Major, and Labrecque 2014). Adventitious root formation may be advantageous for operational‐scale field planting as it allows plants to compensate for root loss due to decay, transform toxins in the rhizosphere to less‐toxic compounds, and produce cytokinins and gibberellins necessary for plant growth (Kozlowski 1992), which could improve establishment success. For example, Mosseler, Major, and Labrecque (2014) tested seven willow species for survival on coal mine sites in common garden field tests and found that wild cuttings of the riparian species *S. eriocephala* and *S. interior* had the highest survival rates. Further, Edward and Cooper (2004) found that willow cuttings with adventitious root formation present had greater survival along a gradient in water table depth due to better tolerance of dry conditions, although root formation may also influence cutting survival by allowing them to withstand prolonged flooding (Talbot, Etherington, and Bryant 1987; Pezeshki, Anderson, and Shields 1998). The greater survival we detected within lowland‐sourced cuttings may result from a greater abundance of riparian species with traits that improve survival post‐planting and along the moisture gradients present in our study.

In a commonly utilized field guide in the region (Johnson et al. 2017), 32 willow species are listed and 16 species are described in detail, which reflect the more common species found in western boreal and aspen parkland. All the willows identified in the present investigation were mentioned ( *S. petiolaris* ) or described (*S. pseudomonticola, S. pseudomyrsinites, S. maccaliana, S* *. discolor* , *S. arbusculoides, S. scouleriana, S. planifolia*, and *S. bebbiana* ) in Johnson et al. (2017). Another government publication for the province of Alberta noted four willows as being the most common regionally, including *S. bebbiana* Sarg., *S. candida* Fluegge ex. Willd., *S. discolor* Muhl., and *S. interior* Nutt (Inkpen and Eyk 1990), with both *S. discolor* and *S. bebbiana* observed in the present investigation. Given that we found ~50% of the same commonly occurring species noted in two sources, it does support the assertion that our sampling was reasonably representative of willows of the region, especially given the relatively small sampling area from which we collected the willow cuttings.

We speculate that the greater survival of larger willow cuttings we detected across source locations may result from greater quantities of growth hormones (e.g., auxin concentration), stored carbohydrates, and available minerals within tissues for adventitious root formation (da Costa et al. 2013). Auxin concentrations, for example, may mobilize carbohydrates in the upper stem for root production and initiate adventitious root production (da Costa et al. 2013; see Haissig 1986), enhancing cutting survival. Woody plants, in general, have greater carbohydrate concentrations in bark, although the total carbohydrates are greater in wood tissue (Pallardy 2008), and larger cuttings with more wood tissues may therefore have greater amounts of carbohydrates. Such size differences may be particularly important for ensuring that cuttings have sufficient carbohydrates following dormancy (Fege and Brown 1984), enhancing root formation and post‐planting survival. Stored carbohydrates may also impact the survival differences we observed among cutting size classes given their importance for cold hardiness and plant defense (see Kozlowski 1992).

### 4.1 Study Limitations

As the original intent of this study was focused on cutting size and success, the original number of cuttings collected per species was not known; therefore, individual species survival success cannot be directly inferred. Rather, we intended to collect cuttings in a manner analogous to operational reclamation practices, where practitioners may have limited knowledge of willow species identification, by collecting cuttings from multiple plants and locations to capture variation in species and genetic diversity. We note that the non‐riparian species *S. bebbiana* dominated surviving cuttings of upland origin (~68%) compared to the riparian species *S. planifolia* (~12%), while *S. planifolia* was most abundant among the surviving lowland cuttings (~42%). *S. bebbiana* is known to have poor rooting ability (Densmore and Zasada 1978; Mosseler, Major, and Labrecque 2014), and very low survival under common garden field planting within reclaimed coal sites has been demonstrated (Mosseler, Major, and Labrecque 2014). Our findings indirectly support this species having a lower capacity for vegetative propagation compared with *S. planifolia* if the proportion of species we identified among cuttings reflects the proportions in original collection areas; however, a comparative trial of these species would be required to validate this observation.

Population‐level differences between sources may be an additional factor driving the observed differences in cutting survival, though the study design limits our ability to assert this as a driving difference between lowland and upland collection sites. Previous work has demonstrated that genetic variability in *Salix* species leads to variance in plant survival under differing environmental conditions (Mosseler and Major 2014; Mosseler, Major, and Labrecque 2014). Additionally, lowland cuttings may be physiologically primed for the stress of planting, as source individuals are likely to have experienced more variable abiotic conditions prior to collection. Future investigations should explore this further through the targeted collection of willows of the same species but growing in varied habitat collecting cuttings from these sources, and then propagating them into a common habitat or ideally, multiple habitats to better assess distinctions between source habitat, establishment habitat, and species‐specific responses.

While not evaluated in the present investigation, there is also some evidence, under controlled conditions, that the position from which cuttings are extracted from parent plants may be an additional factor influencing survival probability (Verwijst et al. 2012). Additional factors that could affect survival and could easily be controlled by practitioners include proper storage and soaking of cuttings prior to planting (Miller‐Adamany, Gerber, and Thomsen 2017).

## 5 Conclusions and Future Research

We found that willow cutting size and source habitat (= species) are pre‐planting sources of variation that alter willow establishment under field‐planting conditions, which may easily be accounted for during operational planning. Based on our findings, we recommend choosing stem cuttings of 50–100 cm length and not less than 2 cm in diameter. Future studies should assess the influence of cutting position on survival probability and the potential factors driving the upland versus lowland differences in cutting survival that we detected in the present investigation. These factors include species‐specific and population‐level differences and physiological priming of cuttings selected from plants growing under more abiotically stressful conditions.

## Author Contributions

**Alan Pollock:** conceptualization, methdology, investigation, data curation, writing – original draft (equal). **Kyle R. Grant:** writing – original draft (equal), visualization. **Amanda Schoonmaker:** conceptualization, investigation, data curation, supervision, project administration, writing – review and editing.

## Conflicts of Interest

The authors declare no conflicts of interest.

## Supporting information


Appendix S1.

Data S1.

## Data Availability

We have provided the raw data from this study as Supporting Information.
