# Supplementary material for: Size Influences on the Survival of Willow Cuttings Under Operational Field Conditions
Source: Ecol Evol. 2025 Jan 9;15(1):e70835. doi: 10.1002/ece3.70835 (PMC11717552; doi:10.1002/ece3.70835)
Supplement: Supplementary file 1 — Appendix S1. [file ECE3-15-e70835-s001.docx]

**Appendix**

**Table A1.** Summary of *Salix* species identified in terms of general plant size and site types / habitats the species is known to occur in. Information adapted from Johnson et al (2017).

| **Species** | **Growth habit** | **Disturbed sites** | **Clearings / forest edges** | **Sand beaches and dunes** | **Open forests** | **Moist forests** | **Lakeshores / riverbanks / floodplains** | **Swamps / wetlands** | **Wetlands** | **Peatlands** | **Fens** | **Sedge meadows** |
| --- | --- | --- | --- | --- | --- | --- | --- | --- | --- | --- | --- | --- |
| *Salix myrtillifolia* var. *cordata* Andersson | 1-4 m tall |  |  |  |  | x | x |  |  | x |  |  |
| *Salix pseudomonticola* C.R. Ball | 1-4 m tall |  | x |  |  |  | x | x | x |  |  |  |
| *Salix petiolaris* Sm. | 0.5-5 m tall |  |  |  |  |  | x |  |  |  |  | x |
| *Salix maccalliana* Rowlee | 1-5 m tall |  |  |  |  |  | x |  | x |  |  |  |
| *Salix discolor* Muhl. | 2-6 m tall |  | x |  | x |  | x | x |  |  | x |  |
| *Salix arbusculoides* Andersson | 1-7 m tall |  |  |  | x |  | x |  | x |  |  |  |
| *Salix scouleriana* Barratt ex Hook. | 2-7 m tall |  |  | x | x |  | x |  |  |  |  |  |
| *Salix planifolia* Pursh | 0.5-4 m tall |  | x |  |  | x | x |  |  |  |  |  |
| *Salix bebbiana* Sarg | 0.5-5 m tall | x |  |  |  |  | x |  | x |  |  |  |

**Table A2.** *Evaluating the effect of plot ID where*: (a) Generalized linear mixed-effects model output for willow cutting survival for cuttings of the 0.5-1.0 cm diameter class. Factors tested included the replicate plot (plot ID) and the size of the cutting (length). Survival data was fit with a binomial distribution. Factors were considered significant at ∝ < 0.05. (b) Estimated marginal mean survival (expressed as a proportion) in year 1 (2013) and year 2 (2014) of willow cuttings collected from lowland and upland locations analysed as a two-factor model where factor 1 was length of cutting and factor 2 was the plot it originated from. Significant main-effect differences amongst means due plot ID (within the year of assessment) are shown by lower-case letters. Values in brackets are 95% confidence intervals on the treatment-level mean (n=21-28 cuttings per plot). NA = mean values were 0 with confidence intervals not estimable due to lack of variation around the mean.

| **Year of assessment** | **Factor level** | **Chi-square value** | **Degrees of freedom** | **p-value** |
| --- | --- | --- | --- | --- |
| Year 1 (2013) | Plot ID | 147.39 | 17 | <0.0001 |
|  | Length | 101.19 | 3 | <0.0001 |
| Year 2 (2014) | Plot ID | 82.433 | 17 | <0.0001 |
|  | Length | 68.553 | 3 | <0.0001 |

| **Borrow pit** | **Plot ID** | **Year 1 (2013)** | **Year 2 (2014)** | **% decline in survival from year 1 to year 2** |
| --- | --- | --- | --- | --- |
| Blue BP | Lowland plot 1 | 0.54bcd (0.42-0.66) | 0.28bcd (0.18-0.40) | 48 |
|  | Lowland plot 2 | 0.31abc (0.18-0.48) | 0.20abcd (0.10-0.36) | 36 |
|  | Lowland plot 3 | 0.45abcd (0.29-0.63) | 0.19abcd (0.08-0.38) | 59 |
|  | Upland plot 4 | 0.36abc (0.24-0.50) | 0.00 (NA) | 100 |
|  | Upland plot 5 | 0.17a (0.09-0.30) | 0.02abc (0.00-0.11) | 90 |
|  | Upland plot 6 | 0.36abc (0.24-0.50) | 0.00 (NA) | 100 |
| Pink BP | Lowland plot 1 | 0.79d (0.67-0.87) | 0.36cd (0.25-0.48) | 55 |
|  | Lowland plot 2 | 0.77d (0.66-0.85) | 0.25abcd (0.17-0.36) | 67 |
|  | Lowland plot 3 | 0.62cd (0.51-0.71) | 0.27bcd (0.19-0.37) | 57 |
|  | Upland plot 4 | 0.59cd (0.48-0.69) | 0.22abcd (0.14-0.31) | 63 |
|  | Upland plot 5 | 0.56bcd (0.45-0.66) | 0.17abc (0.10-0.25) | 70 |
|  | Upland plot 6 | 0.36abc (0.26-0.46) | 0.09ab (0.05-0.16) | 76 |
| Yellow BP | Lowland plot 1 | 0.48abcd (0.35-0.61) | 0.39cd (0.27-0.52) | 19 |
|  | Lowland plot 2 | 0.53bcd (0.40-0.66) | 0.27bcd (0.17-0.39) | 50 |
|  | Lowland plot 3 | 0.74d (0.62-0.84) | 0.45d (0.32-0.58) | 40 |
|  | Upland plot 4 | 0.21a (0.13-0.31) | 0.08ab (0.04-0.16) | 61 |
|  | Upland plot 5 | 0.27ab (0.17-0.40) | 0.05ab (0.01-0.14) | 82 |
|  | Upland plot 6 | 0.20a (0.13-0.31) | 0.03a (0.01-0.10) | 83 |

**Table A3.** *Evaluating the effect of borrow pit location*: (a) Generalized linear mixed-effects model output for willow cutting survival for cuttings of the 0.5-1.0 cm diameter class. Factors tested included the borrow pit location (BP) and the size of the cutting (length). Survival data was fit with a binomial distribution. Factors were considered significant at ∝ < 0.05. (b) Estimated marginal mean survival (expressed as a proportion) in year 1 (2013) and year 2 (2014) of willow cuttings collected from lowland and upland locations analysed as a two-factor model where factor 1 was length of cutting and factor 2 was the borrow pit the plots were established within. Significant main-effect differences amongst means due plot ID (within the year of assessment) are shown by lower-case letters. Values in brackets are 95% confidence intervals on the treatment-level mean (n=21-28 cuttings per plot). NA = mean values were 0 with confidence intervals not estimable due to lack of variation around the mean.

(a)

| **Year of assessment** | **Factor level** | **Chi-square value** | **Degrees of freedom** | **p-value** |
| --- | --- | --- | --- | --- |
| Year 1 (2013) | BP | 147.39 | 17 | 0.0170 |
|  | Length | 101.19 | 3 | <0.0001 |
| Year 2 (2014) | BP | 82.433 | 17 | 0.1323 |
|  | Length | 68.553 | 3 | <0.0001 |

(b)

| **Borrow pit** | **Year 1 (2013)** | **Year 2 (2014)** | **% decline in survival from year 1 to year 2** |
| --- | --- | --- | --- |
| Blue BP | 0.36a (0.24-0.5) | 0.06a (0.02-0.16) | 83 |
| Yellow BP | 0.39ab (0.27-0.53) | 0.15a (0.07-0.32) | 61 |
| Pink BP | 0.62b (0.48-0.74) | 0.21a (0.09-0.4) | 66 |

**Table A4.** Percentage of species identified grouped by borrow pit location.

| ***Salix* species** | **Blue BP** | **Pink BP** | **Yellow BP** |
| --- | --- | --- | --- |
| *S.arbusculoides* | 33 | 2 | 12 |
| *S.bebbiana* | 19 | 37 | 16 |
| *S.discolor* | 4 | 2 | 1 |
| *S.maccalliana* | 7 | 11 | 14 |
| *S.petiolaris* | 0 | 10 | 6 |
| *S.planifolia* | 37 | 24 | 43 |
| *S.pseudomonticola* | 0 | 2 | 3 |
| *S.pseudomyrsinites* | 0 | 10 | 3 |
| *S.scouleriana* | 0 | 5 | 1 |
